# Assessment of Landslide Susceptibility Based on Multiresolution Image Segmentation and Geological Factor Ratings

**DOI:** 10.3390/ijerph17217863

**Published:** 2020-10-27

**Authors:** GongHao Duan, JunChi Zhang, Shuiping Zhang

**Affiliations:** 1 School of Computer Science and Engineering, Wuhan Institute of Technology, Wuhan 430205, China; Duangh@cug.edu.cn (G.D.); zjc.whu@gmail.com (J.Z.); 2 Hubei Provincial Key Laboratory of Intelligent Robots, Wuhan Institute of Technology, Wuhan 430205, China

**Keywords:** landslide, susceptibility, object-oriented, multiresolution segmentation, C5.0 decision tree

## Abstract

Evaluating the susceptibility of regional landslides is one of the core steps in spatial landslide prediction. Starting from multiresolution image segmentation and object-oriented classification theory, this paper uses the four parameters of entropy, energy, correlation, and contrast from remote-sensing images in the Zigui–Badong section of Three Gorges Reservoir as image texture factors; the original image data for the study area were divided into 2279 objects after segmentation. According to the various indicators of the existing historical landslide database in the Three Gorges Reservoir area, combined with the classification processing steps for different types of multistructured data, the relevant geological evaluation factors, including the slope gradient, slope structure, and engineering rock group, were rated based on expert experience. From the perspective of the object-oriented segmentation of multiresolution images and geological factor rating classification, the C5.0 decision tree susceptibility classification model was constructed for the prediction of four types of landslide susceptibility units in the Zigui–Badong section. The mapping results show that the engineering rock group of a high-susceptibility unit usually develops in soft rock or soft–hard interphase rock groups, and the slope is between 15°–30°. The model results show that the average accuracy is 91.64%, and the kappa coefficients are 0.84 and 0.51, indicating that the C5.0 decision tree algorithm provides good accuracy and can clearly divide landslide susceptibility levels for a specific area, respectively. This landslide susceptibility classification, based on multiresolution image segmentation and geological factor classification, has potential applicability.

## 1. Introduction

In the field of geological hazard investigation and research, landslide hazards are a very common geological phenomenon that represents the process and result of sliding downward along a shear plane or a slope [1]. To effectively determine the location of landslide disasters, it is critical to accurately assess the disaster-prone environments of landslides in key research areas [2]. Regional landslide susceptibility research has also been widely performed around the world [3,4].

Landslide susceptibility assessment is the basic task in risk assessment, and it can reduce the difficulty of modeling geological disasters in space. Scholars generally use knowledge-driven models of susceptibility analysis in the engineering field or data-driven models that automatically identify, from trends in data and output, the probability of landslide occurrence in a certain area [5,6,7]. From the perspective of landslide sensitivity, Guzzetti et al. [8] studied the susceptibility of regional landslide groups in Italy and established a theoretical system of landslide risk assessment based on an accuracy assessment of different models. Eeckhaut et al. [9] considered the characteristics of two different quantitative and qualitative models, applied the models in a disaster susceptibility assessment of the landslide group in Champagne Aden Province, and found that the mathematical theory based on a statistical regression model best reflected the actual scenario; notably, the results of landslide area susceptibility zoning were best with this approach. Other scholars have used the texture features of high-resolution remote sensing images to describe spatially stable or unstable areas of landslides in detail [10]. Recently, advancements in remote sensing technology and geographic information systems (GISs) have improved regional stability evaluations of landslides. The former has provided a powerful tool for the spatial identification of unknown landslides and the qualitative analysis of known areas, and the latter provides data support for landslide research and has introduced the concept of data mining to analyze data in landslide areas [11]. Chen et al. [12] combined probability theory and mathematical modeling methods using a population evolution algorithm and neuro-fuzzy system to evaluate and optimize landslide susceptibility from multiple perspectives. It must be noted that the theoretical basis of this type of mathematical model relies on engineering geology analogies and assumes that the geological engineering conditions of the area where landslide disasters may occur are similar to those in areas with landslides [13]. Pourghasemi et al. [14] systematically discussed the accuracy of landslide susceptibility assessment algorithms and found that the current nondeterministic methods may have certain defects. Considering the limitations of a single model and the complexity of landslide hazard evolution by adopting the concept of multimodel fusion, many scholars have researched landslide susceptibility [15,16,17]. The combined multimodel and multifactor landslide susceptibility research methods have achieved some good results, but there are still shortcomings. Many geological datasets have not yet been effectively analyzed, so the mechanism of the occurrence of landslide disasters in some study areas is not fully understood. When evaluating the susceptibility of different locations or types of landslides, the existing modeling methods are likely to fail.

Based on the previous studies above, this paper combines remote sensing factors (entropy, energy, correlation, and contrast) and multidimensional geological and environmental factors (e.g., reservoir water, the bedrock composition, the slope structure) from the perspectives of object-oriented segmentation with multiresolution remote sensing images and geological factor rating classification. In this way, a stable, intelligent, space-division process and corresponding association rules were constructed, and the C5.0 decision tree susceptibility classification model was built. We performed the mapping of landslide susceptibility in the Three Gorges Dam research area.

## 2. Materials and Methods

**Figure 1:**
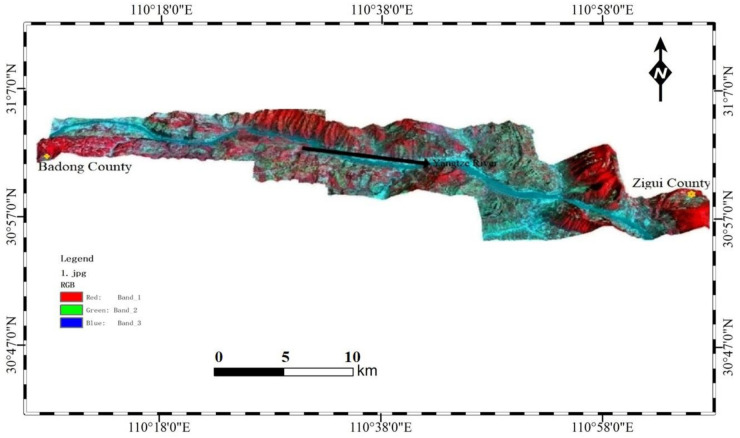
Topographic image of the Zigui–Badong county area (three-dimensional landform overlay Landsat-8 image).

In some reservoir areas around the world, disturbances to the natural bank slope caused by reservoir storage and release are often accompanied by coastal landslide instability, so regional landslides have attracted considerable attention [18]. Our study area is located in the Zigui–Badong section of the Three Gorges Reservoir on the Yangtze River. This section is located in western Hubei, east of Yichang City, Hubei Province. The latitude and longitude are approximately 110°18′–110°52′ east longitude and 30°1′–30°56′ north latitude. The total length of the study region is approximately 55 km, and the entire area is about 446 km^2^. The main terrain includes typical basin topography, and the basin edge is low in the northeast and high in the southwest (Figure 1). In this paper, the decision tree principle and an object-oriented segmentation method for multiresolution remote sensing images are used to process and predict landslide susceptibility in this area.

We collected six scenes of recent Landsat Thematic Mapper (TM) data covering the study area and high-resolution SPOT images with panchromatic band information; these images were mainly used to extract texture features and select samples for remote sensing interpretation. Other data included 1:200,000 scale geological maps, 1:10,000 scale topographic maps, and high-precision DEMs (digital elevation models), which were mainly used to extract relevant topographic and geological factors.

### 2.1. C5.0 Decision Tree Principle

The C5.0 decision tree is a typical classification model. Its binning operation for continuous data is the main operation, making the classification result easy to convert into knowledge [19]. The algorithm uses information entropy as the basic evaluation index, and the best grouping index and segmentation threshold is the information gain so that the training data are classified prior to use based on the data type with the highest information gain. The information gain function is

(1) $$Gain(X)=I(S_{1},S_{2},\cdots ,S_{m})-E(X)$$

where *X* is a data sample, and S represents the m-type samples predivided by *X*. From the above formula, we can determine that the amount of information gain (*Gain*) represents the value of information entropy (*I*) minus conditional entropy (*E*). The core concept of a decision tree is to use top-down and divide-and-conquer recursive functions to form a goal decision tree; thus, this algorithm is a typical greedy algorithm. With the continuous indepth construction of the decision tree, the training sample set is recursively divided into several smaller subsets. This paper combines boosting technology to build a high-precision decision tree model for the generation of association rules for landslide susceptibility mapping in the study area.

### 2.2. Multiresolution Segmentation Concept

**Figure 2:**
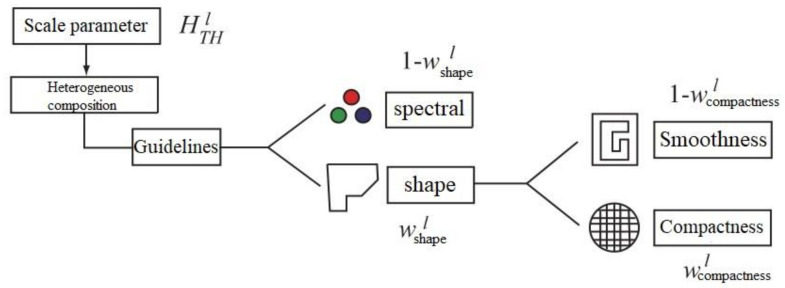
The concept of multiresolution segmentation (revised from the Definiens Developer 7 reference book).

Multiresolution segmentation processing is the core method for the comprehensive extraction of landslide remote sensing data. This method mainly includes two key steps: multiresolution segmentation and object-oriented image classification. By combining object-oriented methods, spatial information such as spectrum, texture, shape, and correlation information from remote sensing images can be used; notably, single or multitemporal spatial data can be fully utilized [20]. A regional merging strategy based on a heterogeneity criterion seeks to minimize the heterogeneity among the weights of image objects after segmentation; before segmentation, it is necessary to determine two factors related to the heterogeneity threshold (Figure 2): the spectral factor and the shape factor. The shape factor is divided into compactness and smoothness.

The segmentation process starts with a single image object (seed), and under the condition of the set homogeneity threshold, the object is enlarged by repeated loop merging. During processing, the seed object is matched with a merged object with the most suitable neighborhood. If the neighboring pixels contain spectral and shape features with minimal heterogeneity, they are merged. If the conditions are not met, a new seed object is established, and pixels that meet the conditions are identified. In a loop, each object is processed only once until no new objects are merged in the image. This object-based segmentation approach has the advantages of high efficiency and good continuity for classification results [21].

## 3. Results

### 3.1. Object-Oriented Segmentation Results in this Research Area

After preprocessing, such as atmospheric correction, cropping, and registration, the existing data processing functions of eCognition software were used to analyze and obtain the spectral and texture characteristics of the Zigui–Badong County images. The texture features of the TM4, TM3, and TM2 bands were selected to reflect the vegetation coverage and landslide morphological characteristics in the study area.

**Figure 3:**
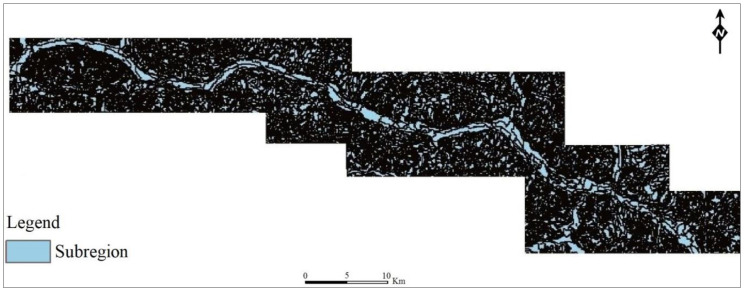
Multiresolution texture segmentation results for Zigui–Badong County (based on Landsat-8 TM4, TM3, and TM2 band data; division into 2279 subregions).

By combining the four parameters (correlation, contrast, energy, and entropy) of the near-infrared, red, and blue bands of the Landsat-8 imagery for the study area as evaluation factors, we strove to evaluate the vegetation coverage and landslide morphological characteristics in the study area as completely as possible. After many repeated verifications of the segmentation results, the scale was set to 30, and the shape heterogeneity parameter was set to 0.4; the results were also optimal when the color heterogeneity parameter was set to 0.6, and the smoothness and compactness factors were all set to 0.5. Finally, the image of the study area was obtained and segmented with this algorithm to obtain 2279 new regions (Figure 3).

### 3.2. Geological Factor Rating

In the past 20 years, comprehensive mathematical modeling and theoretical remote sensing foundations based on mathematical statistical analysis, artificial empirical reasoning, and other nondeterministic prediction methods have become relatively mature [22]. However, there are still inherent defects in these methods, such as strong subjectivity, significant hysteresis, and low model applicability; many models established from spatial factors, so they lack indepth information related to the disaster mechanism for specific types of landslides. In addition to the image texture features, a study of landslide spatial susceptibility should appropriately combine effective environmental and geological factors for targeted analysis. Historical studies have shown that the engineering rock group in a study area highly influences the development of landslides; notably, slippery formations such as mudstone, marl, and siltstone are present in the Badong group. The engineering rock group classification is shown in Table 1.

**Figure 4:**
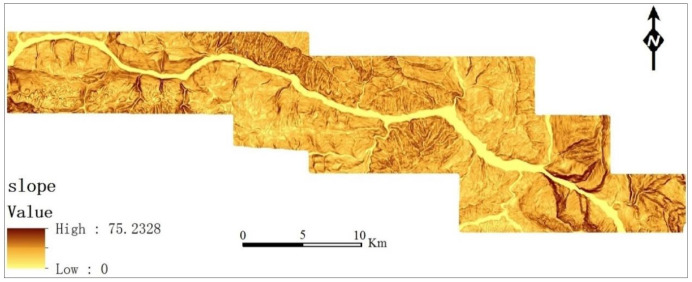
Slope distribution map, with many steep slopes greater than 45° and gentle slopes less than 15°.

In addition, the slope determines the stress distribution characteristics and, to a certain extent, the spatial susceptibility distribution of regional landslides; therefore, it is an important control factor for the development of landslides and needs to be further classified. The slope distribution map for the study area is shown in Figure 4.

The geological factors above were mainly extracted through ArcGIS version 10.2 (Environmental Systems Research Institute, Inc., RedLands, USA) using topographic maps, high-precision DEMs, and geological maps, and the extracted factors were classified. Additionally, the main geological factors that influence the development of landslide spatial stability were extracted with the ArcGIS tool. According to the various indicators of the existing historical landslide database in the Three Gorges Reservoir area, combined with the classification processing steps for different types of multistructured data, the relevant geological evaluation factors were rated based on expert experience, and the resulting geological evaluation factors are shown in Table 2.

### 3.3. Construction of Landslide Susceptibility Model

A thorough investigation of the existing landslide data in the study area revealed that rock and soil landslides are the two main landslide types. Based on the existing research methods for landslide interpretation, the interpretation of the former was based on whether there was an obvious sliding surface, the environmental conditions for disasters, and the impact indicators for reservoir water. The interpretation of the latter was mainly based on an analysis of the slippery rock formations and signs such as reservoir water immersion and slope movement characteristics [23]. In this paper, we integrate the characteristics of geological factor rating indexes to build an intelligent decision tree model for landslide spatial prediction from the Zigui to Badong section of Three Gorges Reservoir. Zoning mapping was performed based on specific classification results, and the accuracy of the model was evaluated.

As noted in Section 3.1, the original image data for the study area were divided into 2279 objects after multiresolution segmentation. Following the principle of uniform coverage, 730 individuals were used as samples, accounting for 32% of the total data. There were 180 landslide areas with an object value of 1, and 550 object attribute values in nonlandslide areas were all assigned a value of 0. Then, we used the IBM SPSS STATISTICS version 23 (IBM SPSS, Inc., Chicago, IL, USA) tool to build the C5.0 decision tree model and implement the classification process based on the decision tree algorithm and 2279 object samples, with 70% used as training samples and 30% used as test samples.

**Figure 5:**
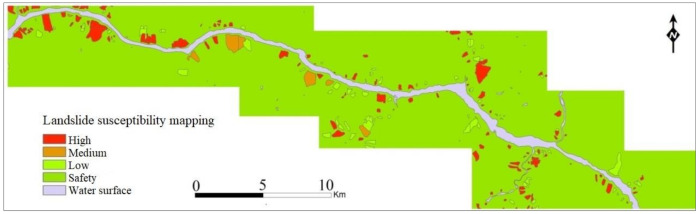
Landslide susceptibility mapping in Zigui–Badong County near Three Gorges Reservoir based on the 4 degrees of spatial development of landslides, the C5.0 decision tree algorithm with image segmentation, and geological factor evaluation, which yields good classification results.

Finally, landslide susceptibility maps were generated according to the above model, as shown in Figure 5. From the prediction results of susceptibility classification, we find that the study area mainly includes safe and high-susceptibility areas. Only 141 low-susceptibility and medium-susceptibility areas are predicted, accounting for 6.19% of all potential areas; the modeling basis is detailed in Section 4.

## 4. Discussion

According to the steps of C5.0 model construction, the classification details for 2279 objects from Zigui to Badong are shown in Table 3 and Table 4, and the results are divided into a training set and a test set.

The results of the C5.0 model have a correct identification rate of 93.73% for the training set and 86.76% for the test set. Thus, the model prediction accuracy is good. The kappa coefficient is used to evaluate the classification accuracy. When the coefficient is greater than 0.4, the classification results have medium or high consistency; the kappa coefficients of the training set and test set were 0.84 and 0.51, respectively, which indicate the excellent performance of the model in classification tasks.

Due to the characteristics of the data sources, the classification results have two formats: one is represented by discrete values of 0 and 1, where 0 represents a stable area and 1 represents an area with a high probability of landslides; the other is a continuous structure. We used values in the interval from 0 to 1 to indicate the influence of each factor on the spatial development of landslides. The larger the value is, the more likely landslides are to occur in the area. From the perspective of landslide susceptibility evaluation, the continuous numerical characteristics are divided into four grades according to the method of natural cracks (Table 5), which is the basis for Figure 5.

Limited by the spatial resolution of the image, traditional remote sensing monitoring and extraction can only rely on the spectral information of the image, and, additionally, the accuracy and efficiency of information extraction cannot be balanced. Therefore, Aksoy and Ercanoglu [24] proposed an object-oriented analysis method, used objects as the basic unit of classification for landslide identification and classification, and improved certain classification accuracy. However, this method cannot fully consider the complex geological information of landslides. Some scholars have made full use of GIS technology and machine learning tools to analyze related influencing factors of landslide susceptibility, and there is no targeted research on the combination of geological factors and images in the Three Gorges Dam research area. Hence, we combined remote sensing factors and multidimensional geological and environmental factors, from the perspectives of object-oriented segmentation, with multiresolution remote sensing images and geological factor rating classification. Finally, the C5.0 decision tree susceptibility classification model was built, and the accuracy evaluation test and landslide susceptibility mapping indicated the excellent performance of the model in classification tasks.

## 5. Conclusions

As the internal and external factors that affect the occurrence of landslide disasters are constantly changing, the current prediction methods have many limitations, and most of the research on the evolution mechanisms of landslides have been performed under relatively limited conditions. Therefore, it is necessary to study the development trends of the geological environment and the evolution of landslide areas in detail to establish methods and models for regional susceptibility evaluation and mapping. In recent years, multisource conversion data and multidirectional monitoring methods have become increasingly abundant [25]. With the continuous development of computer data processing and modeling methods, it has become possible to use multifactor fusion technology and dynamic prediction methods to assess landslide hazard susceptibility.

From the perspective of the object-oriented segmentation of multiresolution images and geological factor rating classification, this paper constructed a C5.0 decision tree model based on image texture factors, reservoir water impact grades, slope gradient, slope structure, and engineering rock group data. We forecasted the susceptibility of four types of stability units in the study area. The average accuracy of the training samples and test sets is 91.64%, and the kappa coefficients are 0.84 and 0.51, respectively, indicating that the C5.0 decision tree provides good accuracy and can clearly divide landslide susceptibility levels for a specific area; the landslide susceptibility map of the study area is established through the decision tree model, and the prediction results show that the high-susceptibility areas are located near the banks of the Yangtze River and its tributaries. Moreover, when the engineering rock group is Jurassic, Silurian strata, or Permian strata, it is likely to be a high-susceptibility slope unit. In summary, based on the geological factor ratings for the study area, the prediction results of the landslide spatial susceptibility model, using multiresolution image segmentation and the C5.0 decision tree model, are reliable.

## Figures and Tables

**Table 1:** Classification of engineering rock groups (Zigui–Badong county).

Major Category	Group	Lithology Description
Carbonate rocks	Karst carbonate rock group (I, II, and III)	Mainly limestone, dolomite, and dolomite limestone
Clastic rocks	Sandstone, argillaceous siltstone interbedded with shale and coal seams, and mudstone shale interbedded rock group (I, II, and III)	Mainly sandstone and argillaceous siltstone interbedded with mudstone or with each other
Carbonate and clastic interbedded rocks	Marl and weak layered siltstone interphase rock group	Limestone, marl, and siltstone; argillaceous siltstone alternating

**Table 2:** Geological evaluation factors.

Evaluation Factor	Group	Description
Reservoir water impact	Weak influence	>430 m
	Intermediate impact	320–430 m
	Strong influence	175–320 m
	Main fluctuation area	145–175 m
Engineering rock group	Mainly hard	Mainly limestone
	Mainly soft	Mainly shale
	Soft and hard	Mainly sandstone
Slope gradient	Ping gentle slope	<15°
	Gently inclined slope	15–30°
	Moderately inclined slope	30–45°
	Steep slope	>45°
Slope structure (slope θ, aspect σ, stratum inclination α, and dip angle β, where Y = |σ−α|)	Light floating slope	0° < Y < 30° or 330° < Y < 360°, β > 10° and θ > β
	Level slope	0° < Y < 30° or 330° < Y < 360°, β > 10° and θ = β
	Dipping slope	0° < Y < 30° or 330° < Y < 360°, β > 10° and θ < β
	Bedding slope	30° < Y < 60° or 300° < Y < 330°
	Transverse slope	60° < Y < 120° or 240° < Y < 300°
	Inverse slope	120° < Y < 150° or 210° < Y < 240°
	Reverse slope	150° < Y < 180° or 180° < Y < 210°
	Massive rock	α and β are null

**Table 3:** Results for the training set.

Accuracy Evaluation			Confusion Matrix Results			Kappa
Correct	479	93.73%		0	1	0.84
Incorrect	32	6.27%	0	381	21	
Total	511	100.0%	1	11	98	

**Table 4:** Results for the test set.

Accuracy Evaluation			Confusion Matrix Results			Kappa
Correct	190	86.76%		0	1	0.51
Incorrect	29	13.24%	0	128	20	
Total	219	100.0%	1	9	62	

**Table 5:** Landslide susceptibility classification prediction (Zigui–Badong).

	Forecasting	Forecast Group	Number	Percentage (%)
Discrete data	0	Stable zone	1977	86.75
	1	Risky zone	302	13.25
Continuous data	[0, 0.261)	Safety	1862	81.70
	[0.261, 0.420)	Low	72	3.16
	[0.420, 0.569)	Medium	69	3.03
	[0.569, 1]	High	276	12.11

## References

[1] Nemčok A., Pašek J., Rybář J. (1972). Classification of landslides and other mass movements. Rock Mech. Rock Eng..

[2] Baldo M., Bicocchi C., Chiocchini U., Giordan D., Lollino G. (2009). LIDAR monitoring of mass wasting processes: The Radicofani landslide, province of Siena, Central Italy. Geomorphology.

[3] Zhao B., Wang Y., Wang Y., Feng Q., Li J., Zhao X. (2018). Triggering mechanism and deformation characteristics of a reactivated ancient landslide, Sichuan Province, China. Landslides.

[4] Arabameri A., Saha S., Roy J., Chen W., Blaschke T., Bui D.T. (2020). Landslide susceptibility evaluation and management using different machine learning methods in The Gallicash River Watershed, Iran. Remote Sens..

[5] Arabameri A., Pradhan B., Rezaei K., Lee C.-W. (2019). Assessment of landslide susceptibility using statistical- and Artificial Intelligence-based FR–RF integrated model and multiresolution DEMs. Remote Sens..

[6] Roy J., Saha S., Arabameri A., Blaschke T., Bui D.T. (2019). A novel ensemble approach for landslide susceptibility mapping (LSM) in Darjeeling and Kalimpong districts, West Bengal, India. Remote Sens..

[7] Broeckx J., Vanmaercke M., Duchateau R., Poesen J. (2018). A data-based landslide susceptibility map of Africa. Earth-Sci. Rev..

[8] Guzzetti F., Reichenbach P., Ardizzone F., Cardinali M., Galli M. (2006). Estimating the quality of landslide susceptibility models. Geomorphology.

[9] Eeckhaut M.V.D., Marre A., Poesen J. (2010). Comparison of two landslide susceptibility assessments in the Champagne–Ardenne region (France). Geomorphology.

[10] Nichol J.E., Shaker A., Wong M.-S. (2006). Application of high-resolution stereo satellite images to detailed landslide hazard assessment. Geomorphology.

[11] Huang H., Song K., Yi W., Long J., Liu Q., Zhang G. (2018). Use of multi-source remote sensing images to describe the sudden Shanshucao landslide in the Three Gorges Reservoir, China. Bull. Int. Assoc. Eng. Geol..

[12] Chen W., Panahi M., Tsangaratos P., Dou J., Ilia I., Panahi S., Li S., Jaafari A., Bin Ahmad B. (2019). Applying population-based evolutionary algorithms and a neuro-fuzzy system for modeling landslide susceptibility. Catena.

[13] Okamoto T., Matsuura S., Larsen J.O., Asano S., Abe K. (2018). The response of pore water pressure to snow accumulation on a low-permeability clay landslide. Eng. Geol..

[14] Pourghasemi H.R., Yansari Z.T., Panagos P., Pradhan B. (2018). Analysis and evaluation of landslide susceptibility: a review on articles published during 2005–2016 (periods of 2005–2012 and 2013–2016). Arab. J. Geosci..

[15] Raspini F., Bianchini S., Ciampalini A., Del Soldato M., Montalti R., Solari L., Tofani V., Casagli N. (2019). Persistent scatterers continuous streaming for landslide monitoring and mapping: the case of the Tuscany region (Italy). Landslides.

[16] Tao Z., Wang Y., Zhu C., Xu H., Li G., He M. (2019). Mechanical evolution of constant resistance and large deformation anchor cables and their application in landslide monitoring. Bull. Int. Assoc. Eng. Geol..

[17] Huang Y., Zhao L. (2018). Review on landslide susceptibility mapping using support vector machines. Catena.

[18] Duan G., Chen D., Niu R. (2019). Forecasting groundwater level for soil landslide based on a dynamic model and landslide evolution pattern. Water.

[19] Karlson M., Ostwald M., Reese H., Sanou J., Tankoano B., Mattsson E. (2015). Mapping tree canopy cover and aboveground biomass in sudano-sahelian woodlands using Landsat 8 and random forest. Remote Sens..

[20] Carleer A.P., Wolff E. (2006). Urban land cover multi-level region-based classification of VHR data by selecting relevant features. Int. J. Remote Sens..

[21] Kurtz C., Passat N., Gançarski P., Puissant A. (2010). Multi-resolution region-based clustering for urban analysis. Int. J. Remote Sens..

[22] Pradhan A.M.S., Kang H.-S., Lee J.-S., Kim Y.-T. (2017). An ensemble landslide hazard model incorporating rainfall threshold for Mt. Umyeon, South Korea. Bull. Int. Assoc. Eng. Geol..

[23] Arabameri A., Lee S., Tiefenbacher J.P., Ngo P.T. (2020). T Novel ensemble of MCDM-Artificial Intelligence techniques for groundwater-potential mapping in arid and semi-arid regions (Iran). Remote Sens..

[24] Aksoy B., Ercanoglu M. (2012). Landslide identification and classification by object-based image analysis and fuzzy logic: An example from the Azdavay region (Kastamonu, Turkey). Comput. Geosci..

[25] Erener A.H., Düzgün S.B. (2010). Improvement of statistical landslide susceptibility mapping by using spatial and global regression methods in the case of More and Romsdal (Norway). Landslides.

